# Evaluation of the utility of a rapid test for syphilis at a sexually transmitted disease clinic in Buenos Aires, Argentina

**DOI:** 10.1038/s41598-018-25941-4

**Published:** 2018-05-15

**Authors:** Lucía Gallo Vaulet, Nicolás Morando, Ricardo Casco, Asunta Melgar, Silvia Nacher, Marcelo Rodríguez Fermepin, María A. Pando

**Affiliations:** 1 Universidad de Buenos Aires, Facultad de Farmacia y Bioquímica, Departamento de Bioquímica Clínica, Cátedra de Microbiología Clínica, Inmunología y Virología Clínica, Buenos Aires, Argentina; 2 Universidad de Buenos Aires, Instituto de Fisiopatología y Bioquímica Clínica (INFIBIOC), Buenos Aires, Argentina; 3 CONICET-Universidad de Buenos Aires, Instituto de Investigaciones Biomédicas en Retrovirus y Sida (INBIRS), Buenos Aires, Argentina; 4 Universidad de Buenos Aires, Hospital de Clínicas “José de San Martín”, Programa de Enfermedades de Transmisión Sexual, Buenos Aires, Argentina

## Abstract

Even though syphilis can be easily diagnosed by simple and low-cost laboratory methods, it continues to be an important health problem. Rapid tests (RT) for the detection of treponemal antibodies can facilitate earlier diagnosis, access to treatment and linkage to care. The aim of this study was to analyse the usefulness of the incorporation of a RT in the detection of patients infected with *T. pallidum* in a sexually-transmitted disease (STD) clinic. Between March and December 2015, a syphilis RT was offered to patients who spontaneously attended the clinic. Conventional serology testing was additionally indicated to every participant. The RT for syphilis was offered to 1887 patients, of whom 31.1% agreed to get tested. VDRL test was performed in 84.0% of patients that were also tested with syphilis RT, with a significantly higher frequency observed among participants with reactive RT (94.3% vs. 79.8%, p < 0.001). These results showed that 33.7% of the participants were reactive for the RT and 27.0% were reactive for the VDRL test. Both tests were reactive in 24.9% and non-reactive in 64.3%. A high prevalence of active syphilis was detected in patients attending the clinic. The use of a syphilis RT had a positive impact, which in combination with the VDRL test increased the number of patients that were effectively diagnosed.

## Introduction

Syphilis is a sexually-transmitted disease (STD) caused by the spirochaete *Treponema pallidum* subspecies *pallidum* (*T. pallidum*). Most infected individuals are unaware of their infection while transmission to their sexual contacts effectively occurs. When this infection goes undiagnosed and untreated, it can lead to long-term development of serious manifestations in the cardiovascular system (cardiovascular syphilis), the central nervous system (neurosyphilis), as well as granulomatous lesions (syphilitic gumma)^1,2^. Moreover, infected pregnant women can transmit *T. pallidum* to the foetus leading to serious consequences like intrauterine foetal death, neonatal death and congenital syphilis^3^. In addition, syphilis can cause genital ulcers, which have been associated with an increase in HIV transmission risk^2,4^. Therefore, early detection and treatment are fundamental in the prevention and control of syphilis and serious long-term complications in persons with syphilis as well as congenital syphilis.

Syphilis can be diagnosed by a combination of simple and low-cost laboratory methods and it has been efficiently treated since the discovery of penicillin at the beginning of the 20th century^2^. However, it continues to be an important health problem. According to the World Health Organization, Latin America and the Caribbean is the region with the highest incidence of syphilis worldwide, concentrating three of 12 million new infections^5^.

The global prevalence of syphilis in Argentina is unknown but. However, several studies conducted within the country found a high prevalence among vulnerable populations. Among men who have sex with men (MSM) in Buenos Aires City, the prevalence of syphilis ranged from 16.9% to 20.5%^6,7^, whereas 22.4% of syphilis infection was detected among female sex workers in eight major cities of Argentina^8^ and a 50% prevalence was found among female transgender sex workers in the City of Buenos Aires^9^.

The detection of non-treponemal (anti-cardiolipin antibodies) and treponemal antibodies is still the mainstay of syphilis diagnosis. Treponemal antibodies can be detected in all the stages of syphilis and are used mainly to confirm the infection. On the other hand, non-treponemal antibodies are used in syphilis screening to monitor the status of the infection as well as the response to antibiotic treatment^10^.

Recently, syphilis rapid tests (RT) for the detection of treponemal antibodies have become commercially available in Argentina. This kind of RT can expand the range of settings in which syphilis testing can be undertaken, thus facilitating earlier diagnosis, access to rapid treatment and linkage to care^10,11^. Nevertheless, there is still no local experience on its use and on its usefulness for the detection of infected patients in need of treatment.

The sexually-transmitted disease clinic at the University Hospital (*Hospital de Clínicas “José de San Martín”, Programa de Enfermedades de Transmisión Sexual, PETS*) receives approximately 250 patients’ consultations per month, 45% of whom are MSM. A year previous to this study, syphilis was the second most prevalent infectious disease detected among patients attending the clinic, accounting for 11.4% of all detected infections, after human papillomaviruses (18%) (Ricardo Casco, personal communication). The aim of this study was to analyse the usefulness of the incorporation of a syphilis RT for the detection of patients infected with *T. pallidum* by its implementation in this STD clinic.

## Results

### Characteristics of the study group

**Figure 1:**
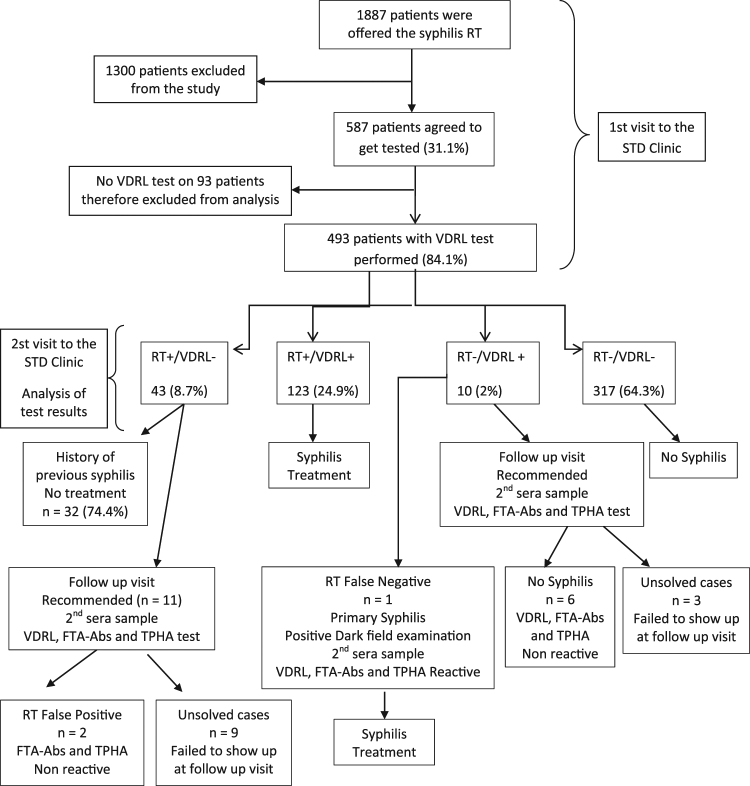
Flowchart showing the flow of participants attending the STD clinic in Buenos Aires, Argentina (2015). Sample size for each step throughout the study is shown.

During the study period, RT for syphilis was offered to 1887 patients who spontaneously attended the STD clinic, whereas 587 of them agreed to get tested (31.1%) (Fig. 1). Table 1 shows socio-demographic characteristics of the study population. Briefly, participants’ median age was 29 years and 78.6% of them were male. Most participants resided in Buenos Aires City (72.2%) or its surroundings in the Buenos Aires Province, whereas participants from other provinces and foreigners were rare. Seventy-five percent of the study population had completed high school and less than 40% reported having a steady job position. According to the participant’s medical records, around 78% of them reported having an appointment with a physician in the previous year. In relation to the type of partner, 96.9% of the female participants reported having male partners. However, 50.2% of the men reported having male partners. Condom use was mostly irregular, with only 26.2% of the study participants reporting regular use.

**Table 1:** Socio-demographic characteristics of 586 participants attending the STD clinic in Buenos Aires, Argentina (2015).

	Total N = 586	With VDRL test N = 493	Without VDRL test N = 93
**Median age (IQR)**	29 (24–38)	29 (24–37)	30 (24–40)
**Gender % (n/N)**			
Male	78.6% (456/580)	79.5% (388/488)	73.9% (68/92)
Female	21.4% (124/580)	20.5% (100/488)	26.1% (24/92)
**Residence**			
City of Buenos Aires	72.2% (345/478)	72.0% (293/407)	73.2% (52/71)
Outside the city	27.8% (133/478)	28.0% (114/407)	26.8% (19/71)
**Formal Education level**			
Complete high school	75.0% (436/581)	74.0% (362/489)	80.4% (74/92)
Incomplete high school	25.0% (145/581)	26.0% (127/489)	19.6% (18/92)
**Employment status**			
Have an steady job	38.9% (218/561)	37.2% (175/470)	47.3% (43/91)
Unsteady job or unemployed	61.1% (343/561)	62.8% (295/470)	52.7% (48/91)
**Last visit to a doctor**			
<1 year	78.5% (427/544)	79.3% (363/458)	74.4% (64/86)
>1 year	21.5% (117/544)	20.7% (95/458)	25.6% (22/86)
**Type of sex partners in the last six months**			
Men (n = 434)			
Only women	49.8% (216/434)	47.0% (174/370)	65.6% (42/64)^*^
Only men or men and women	50.2% (218/434)	53.0% (196/370)	34.4% (22/64)
Women (n = 118)			
Only men	96.9% (114/118)	95.8% (91/95)	100% (23/23)
Only women or men and women	3.4% (4/118)	4.2% (4/95)	0% (0/23)
**Condom use**			
Regular	26.2% (144/550)	26.5% (123/465)	24.7% (21/85)
Irregular	73.8% (406/550)	73.5% (342/465)	75.3% (64/85)

Stratification by VDRL test done.

^*^p < 0.05.

### Analysis of RT results

Of the 587 participants recruited in this study, 493 (84.1%) attended the university hospital laboratory for VDRL testing, with this frequency being significantly higher among participants with reactive RT (94.3% *vs*. 79.8%, p < 0.001).

The analysis of the RT and VDRL results showed that 33.7% (166/493) of participants turned out to be reactive for the RT and 27.0% (133/493) were reactive for VDRL, with both tests being reactive in 123 cases (24.9%). A total of 317 (64.3%) participants were found to be non-reactive in both tests. RT and VDRL test results are summarized in Table 2 and Fig. 1.

**Table 2:** Syphilis Rapid Test and VDRL test results in 493 participants attending the STD clinic in Buenos Aires, Argentina (2015).

VDRL	Reactive RT N (%)	Non-reactive RT N (%)	Total N (%)
Reactive	123 (24.9)	10 (2.0)	133 (27.0)
Non-reactive	43 (8.7)	317 (64.3)	360 (73.0)
Total	166 (33.7)	327 (66.3)	493

In 43 participants, a reactive RT but a non-reactive VDRL test was obtained, 32 of whom (74.4%) had a previous history of syphilis infection stated in their medical records. Of the 11 participants that did not have a recorded history of syphilis infection, two were considered to be false-positive in the RT because treponemal antibodies were not detected by the indirect immunofluorescence test (FTA-Abs) and the treponemal hemagglutination assay (TPHA), leaving nine cases unsolved, given the fact that the participants did not return to the STD clinic for their follow-up.

In a total of 10 participants a reactive VDRL test but a non-reactive RT was obtained. In these cases, VDRL titres ranged from one to four. In six of these cases, syphilis diagnosis was excluded as a non-reactive result was obtained in the VDRL, FTA-Abs and TPHA tests in a second serum sample obtained during the follow-up visit. Nevertheless, one of these participants was diagnosed with primary syphilis infection as revealed by positive dark-field microscopy obtained from material of a lesion. In this case, the non-reactive RT was considered as a false-negative result. The three remaining cases could not be elucidated since participants failed to attend a follow-up visit. Reasons for not returning to the follow-up visit or not getting the VDRL test are unknown. During this study, no further changes were introduced in the standard of care, and patients were free to decide what test they would get and whether they would return to the clinic for further consultation. Comparing socio-demographic characteristics of the study population stratified by VDRL test done or not done, we identified that men who have female partners attend the clinic for a follow-up less frequently than those men who have male partners (Table 1).

### Syphilis prevalence

Syphilis prevalence according to the syphilis RT results, which detects both current and past infection, was 33.7% (166/493). Nevertheless, prevalence of active syphilis, i.e. participants with both reactive RT and VDRL test, was 24.9% (123/493) (Table 1). This prevalence was significantly higher when compared to that of the previous year (11.4%, 156/1364, p < 0.001). All patients with active syphilis received treatment for free and were followed up using VDRL.

## Discussion

Several cheap and simple rapid tests for syphilis are available worldwide and represent an opportunity to expand syphilis diagnosis in hard-to-reach populations who are less likely to return to care. Moreover, it has been reported that the sensitivity of the syphilis RT varies between 60–89% when using whole blood while specificity of the RT were higher than 98%^11^. However, international literature agrees with the need for additional clinical, performance and cost effectiveness assessments in relation to the implementation of RT in different settings^10^.

This study reports, for the first time in Argentina, the usefulness of the RT performed within an STD clinic on detecting patients infected with *Treponema pallidum*. Before this study, the STD clinic’s standard of care was to screen the patients’ lesions for syphilis by dark-field microscopy when present while offering the non-treponemal antibody test to all of them. In 2014, the year prior to that of the current study, syphilis prevalence was 11.4%, using the standard of care. During the study period, the RT was offered to every patient attending the STD clinic but only 31.1% agreed to get tested. Frequent reasons for not getting tested were ‘I am on treatment for syphilis’, ‘I already had syphilis’, ‘The visit to the STD clinic was to retrieve syphilis test results’ and ‘I want to discuss RT for syphilis with a physician first’. Nevertheless, a high prevalence of active syphilis was detected (24.9%) combining VDRL and RT results, and a positive effect of the rapid testing was observed in participants with reactive RT, since they were tested by VDRL more often than participants with non-reactive RT (93.4% *vs*. 79.8%, p < 0.001). This might be due to the physician’s strong recommendation to perform VDRL test on participants with positive syphilis RT and no history of syphilis in their medical records. Also, having to wait for blood extraction at the lab might play a role in the participant’s decision. Therefore, in this context, the introduction of RT together with clinical evaluation and VDRL test increased the diagnostic opportunity of syphilis and the establishment of an effective treatment with penicillin as all positive patients were treated for free in the STD clinic.

The rapid turnaround time for results (15 minutes), the type of specimen used (fingerstick whole blood), and the fact that it can be performed and interpreted by personnel with basic training in testing procedures and biosafety measures are some of the advantages of the RT. Nevertheless, it has been suggested that due to the fact that this type of test can be performed by non-laboratory experienced personnel, the interpretation of the results can vary with the subjectivity of the operator. Moreover, variability among test lots, day-to-day interpretation and differences between operators have been documented^10–13^. In addition, sensitivity variations of the syphilis RT were observed in several studies using whole blood^11,12^ but in our study, a low number of false-positive cases was detected using whole blood on site. To reduce bias, only two experienced lab operators who received specific training on test procedures by trainers from the National Ministry of Health performed and interpreted all the tests and no invalid tests were obtained. Treatment of all RT positive individuals will result in over-treatment, mainly due to the fact that the RT detects treponemal specific antibodies, and thus it cannot distinguish between active and past treated infection, and to a lesser extent, to the presence of false-positive RT results. However, given the serious consequences of missed treatment, in high prevalence settings with poor linkage to health care, the benefits of treatment far outweigh the cost of over-treatment. To hamper this situation, the VDRL test was recommended to every patient in our study and approximately a quarter of the participants with reactive RT (43/166) did not have active syphilis and in their medical records stated history of syphilis and treatment. The decision to refer or not to immediate treatment was made by the physician based on the combination of the participant’s STD history, clinical examination and VDRL test results. Treatment with penicillin is offered and administered for free at the STD clinic, and in a handful of cases, treatment was discontinued upon obtaining the VDRL test result. Furthermore, in this group, only two false-positive RT were detected as non-treponemal antibodies by VDRL test and treponemal antibodies by TPHA, and FTA-Abs were non-reactive.

On the other hand, in a few participants with a non-reactive RT (3.1%, n = 10), a reactive VDRL test was obtained with titres ranging from one to four. In this group, syphilis was discarded in six cases because treponemal antibodies were not detected (neither by TPHA nor by FTA-Abs test). False-positive VDRL test results can be attributed to the presence of anti-cardiolipin antibodies that are not related to syphilis infection but present in other infectious diseases (e.g. malaria, leprosy, Chagas disease, tuberculosis and hepatitis C, among others), or other conditions (autoimmune diseases, pregnancy, old age or malignant diseases)^10,14^. Even more importantly, we identified a case of primary syphilis in a participant with a lesion in the genital region compatible with a syphilitic chancre which had not been detected by RT. In this case, non-treponemal and treponemal antibodies were detected by VDRL test and FTA-Abs test, respectively. Thus, it was considered false negative by RT. This patient was treated with penicillin and VDRL tests were used to monitor treatment efficacy. Our results are concordant with previous studies, suggesting that RT can be used in syphilis diagnosis with similar sensitivity and specificity to other treponemal tests^11,12^.

A limitation of our study was that 11 patients failed to return to the STD clinic for follow-up; thus, a second serum sample was not obtained to solve their syphilis status. Loss in follow-up visit might be due to the fact that VDRL test results were available within 24 h. This issue could be addressed if VDRL tests were performed within the STD clinic but this is not possible in our setting as no laboratory personnel or facilities are available. The dual non-treponemal/treponemal RT might help elucidate these cases and in the treatment initiation decision-making. Most studies conclude that dual detection of non-treponemal and treponemal antibodies can help detect syphilis cases in resource-limited settings while substantially reducing overtreatment rates^15,16^ but the major limitation of this dual non-treponemal/treponemal RT is its low sensitivity shown for low-titre non-treponemal antibodies^16–18^. High sensitivity (98.8%) of this particular RT was observed in a recent study comparing to VDRL tests but limitations were that all tests were performed on serum samples and inside conventional laboratory and no biological false-positive samples were tested^19^. This type of RT that detects treponemal and non-treponemal antibodies simultaneously is not available in our country yet, and lab performance and field evaluations of this dual point-of-care test are still limited.

In conclusion, a high prevalence of syphilis was detected in patients attending the STD clinic of the university hospital, and a low number of false-positive and false-negative RT results was detected in this study. In our setting, the use of the RT impacts positively on the diagnosis of syphilis, increasing the treatment opportunity of positive patients within the STD clinic. Interventions using RT could provide an opportunity to reduce syphilis prevalence, especially in other contexts such as in at-risk populations with documented high prevalence or no linkage to health care (e.g. female-, male- or trans-sexual workers) or in prevention strategies aimed at avoiding vertical transmission.

## Methods

### Study population

Between March and December 2015, RT for syphilis was offered to every patient who spontaneously attended the STD clinic of the University Hospital “*Hospital de Clínicas, José de San Martín*”, *Universidad de Buenos Aires*. Patients who agreed to get tested provided their written informed consent and answered a self-administered questionnaire that included several socio-demographic characteristics such as age, gender, nationality, level of educational attainment and risk factors for sexually-transmitted diseases such as frequency of condom use. Additionally, participants’ medical records were reviewed in order to gather data concerning reason for consultation, history of syphilis and final clinical diagnosis at recruitment. Patients who refused to get tested did not answer the self-administered questionnaire and therefore were excluded from the study. No alterations were introduced to the patient’s standard of care other than the incorporation of the syphilis RT.

### Ethics Statement

International and national ethical guidelines for biomedical research involving human subjects were followed. This study was approved by the University Hospital’s Institutional Review Board (IRB) (*Comité de Ética, Hospital de Clínicas* “*José de San Martín*”, *Universidad de Buenos Aires)*. The work described was carried out in line with The Code of Ethics of the World Medical Association (Declaration of Helsinki) for experiments involving humans. Informed consent was obtained from each participant.

### Study procedures

Due to the fact that the RT was used for the first time in the clinic, prior to the initiation of the study a single theoretical and practical training session in the use of the RT for the clinic staff and researchers involved was carried out by a team of specialists from the National Ministry of Health.

All the individuals who agreed to participate in this study were tested prior to a medical consultation in a separate office within the clinic by a trained health professional that received the complete self-administered questionnaire and explained the test procedures. Fingerstick RT for syphilis was performed on whole blood (Alere Determine Syphilis TP) according to the manufacturer´s instructions. The immunochromatographic test was used to detect specific antibodies against *Treponema pallidum* antigens. These antibodies are present in individuals with active, past or treated infection. Participants were informed that the results would be explained during the medical consultation and that further conventional serology tests for syphilis were likely to be recommended by the physician. Test results were available within 15 minutes and linked to the questionnaire by a unique numeric code preserving the participant’s confidentiality.

After the clinical evaluation, every participant was asked to go to the university hospital´s laboratory in order to obtain a sample of non-anticoagulated blood in sterile conditions using standard safety precautions and protocols. From this sample, non-treponemal antibodies were detected using the Unheated Serum Reagin (USR) test (Wiener Laboratorios, SAIC, Rosario, Argentina) in every participant. The USR test detects anti cardiolipin antibodies (non-treponemal antibodies) in non-inactivated serum samples and is performed and interpreted as a Venereal Disease Research Laboratory (VDRL) test. In the case of discordant results, treponemal antibodies were detected by an indirect immunofluorescence test (FTA-Abs, InmunofluorBiocientifica SA, Argentina) and by *Treponema pallidum* hemagglutination assay (TPHA, BioSystems SA, Barcelona, Spain) in a second serum sample obtained at a follow-up visit at the STD clinic.

### Statistical analyses

Baseline characteristics were described using medians and interquartile ranges (IQRs) for continuous variables, and counts and percentages for categorical data. Comparisons between proportions were analysed by parametric and non-parametric methods. Chi-square and Fisher’s exact tests were used to compare differences in categorical variables. Statistical analyses were carried out using IBM SPSS Statistics Base 22.0 (IBM Corp. Released 2013. IBM SPSS Statistics for Windows, Version 22.0. Armonk, NY: IBM Corp.).
